# Supplementary material for: Comprehensive Annotation and Functional Exploration of MicroRNAs in Lettuce
Source: Front Plant Sci. 2021 Dec 24;12:781836. doi: 10.3389/fpls.2021.781836 (PMC8739914; doi:10.3389/fpls.2021.781836)
Supplement: Supplementary file 2 [file Data_Sheet_8.ZIP › GSM2230749.plot/Lsa-miR390_Lsat_1_v5_gn_7_107941.1_389_TPlot.pdf]

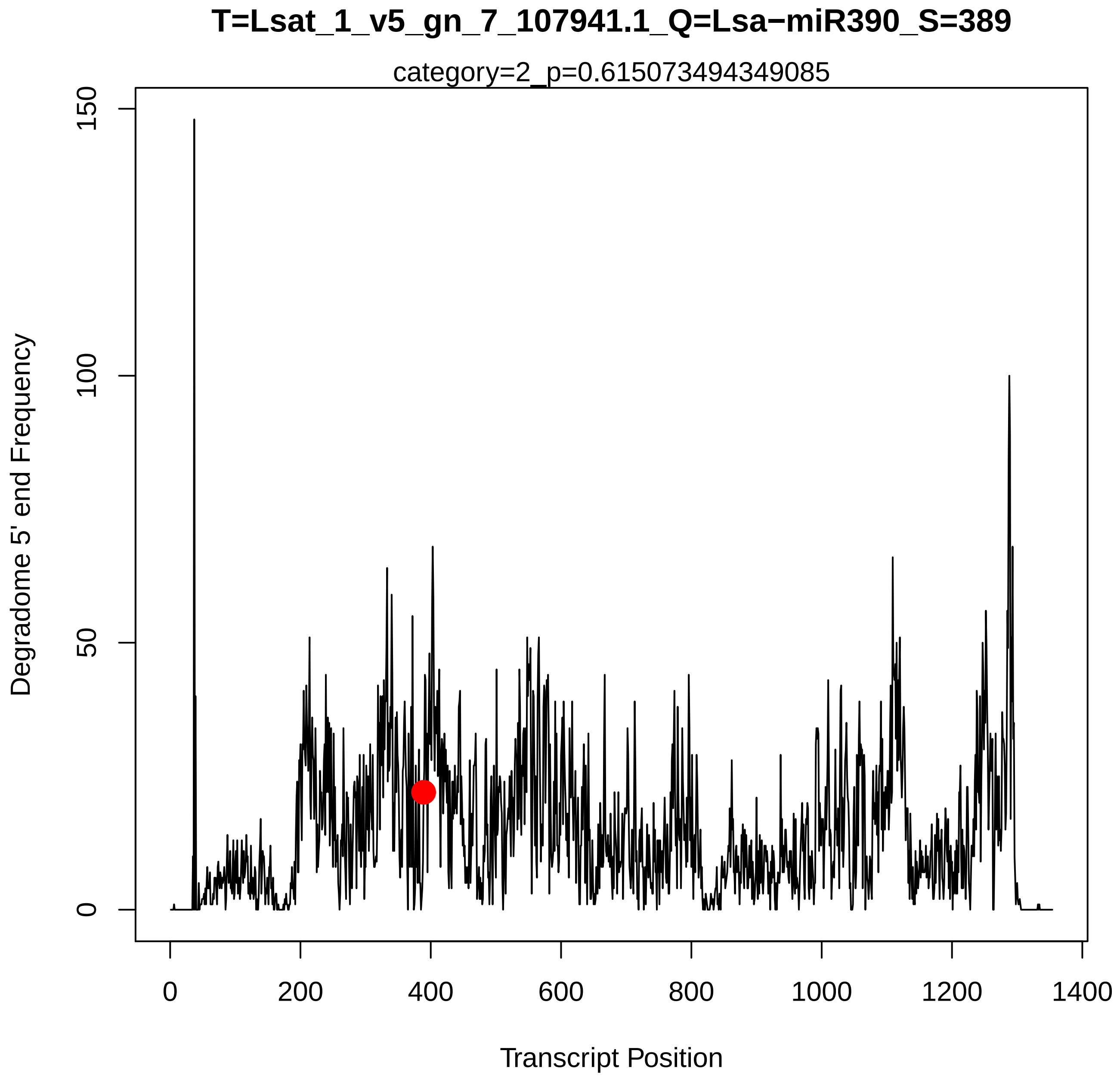

T=Lsat_1_v5_gn_7_107941.1_Q=Lsa−miR390_S=389
category=2_p=0.615073494349085
Degradome 5' end Frequency
0
50
100
150
0
200
400
600
800
1000
1200
1400
Transcript Position
